# School nurses’ engagement and care ethics in promoting adolescent health

**DOI:** 10.1177/0969733020985145

**Published:** 2021-03-03

**Authors:** Yvonne Hilli, Gunnel Pedersen

**Affiliations:** 1786 Nord University, Norway; Sjöbo Health Care Centre, Sweden

**Keywords:** Adolescents, care ethics, caring relation, health promotion, school nurse

## Abstract

**Background:**

The school is a key environment for establishing good health habits among pupils. School nurses play a prominent role in health promotion, since they meet with every single adolescent.

**Research aim:**

To describe care ethics in the context of school nurses’ health-promoting activities among adolescents in secondary schools.

**Research design:**

An explorative descriptive methodology in which semi-structured interviews were used to collect data and content analysis was performed.

**Participants and research context:**

Data were collected from eight school nurses in a municipality in Western Sweden.

**Ethical considerations:**

This study was conducted according to the ethical principles of the Swedish Research Council (2011), and the written informed consent of the participants was obtained.

**Findings/discussion:**

A caring relation, based on care ethics, is the basis for successful health-promoting activities among adolescents. The school nurses show strong engagement in and commitment to caring for and caring about adolescents by being attentive and listening to their expressed feelings and needs, both spoken and unspoken. Furthermore, the school nurses have a deep sense of responsibility in supporting and empowering adolescents to trust their own capabilities. To enhance health and well-being, school nurses emphasize low-threshold counselling, flexibility, openness, early intervention and continuity, as well as good collaboration with the health team at school and with parents.

**Conclusion:**

Strengthening person-centred healthcare can provide adolescents with the recognition and support they need to grow into healthy adults. For successful health promotion, all aspects of the ethics of care should be considered as part of an integrated whole based on the integrity of care.

## Introduction

Adolescents are generally considered to have good health, despite certain differences. However, the older children get, the lower is their estimated health.^1^ The Public Health Agency of Sweden^2^ reported better lifestyle among adolescents, but the incidence of sleep problems, stomach pain, headaches, stress and depression has increased. Importantly, the percentage of 13-year-olds who feel stressed has doubled during the last decade.^1,3^ The school is a key environment for health-promoting work, as good health habits can be established at school.^1,4,5^ The foremost goal of the WHO Health 2020 is to significantly improve health and well-being, and to this end, four areas of priority are emphasized: investment in health through a life course approach that includes empowerment, prevention of major diseases, strengthening person-centred health systems, and creation of supportive and resilient environments. Accordingly, medical interventions, including healthcare, for pupils is provided by doctors and school nurses who have specialized in public health or school nursing.

School nurses in Sweden play a prominent role in health promotion, since they meet with every single pupil. School nurses are expected to have a respectful and empathetic approach to promoting self-care among pupils, and they are required to identify individual needs, conduct assessments and design interventions to support health and well-being.^6^ School healthcare is based on a basic programme that offers health counselling, vaccinations and health dialogues, as well as health education and screening of the back, sight, hearing and colour vision. During elementary school, all pupils are offered three health visits to the school nurse: during pre-school with the parents, in the fourth grade and in the seventh or eighth grade.^7^ The health visit includes a health dialogue, with the aim of early identification of health problems or symptoms. School-age children are interested in their own development and health and, therefore, the school is a suitable environment for health-promoting activities to establish healthy habits.^8^ Beyond the planned health visits, school nurses are available for adolescents during certain hours and days of each week.

## Theoretical perspective

The starting point of this study is caritative caring, which is based on the idea that ‘care’ and ‘caring’ form the essence of nursing.^9,10^ Caring is a core concept of this theory, and the basic motive of caring is to alleviate suffering and to serve life and health. Health promotion is defined as ‘a process of enabling people to increase control over, and to improve, their health’.^1^ To *care for* means that the core of care ethics, the ethos, is present, and this encompasses the basic values.^11,12^ The nature of the caring relationship is determined by the carers’ ethical foundation, their motives for caring and responsibility, and their will and interest in inviting a person into a caring relationship. A carer who is sensitive to the voice of the heart metaphorically experiences at-homeness and has the courage to take on responsibility and be engaged. The ethos becomes evident in the ethical conduct and manner of being. Moreover, the ethos is reflected in the physical room and atmosphere that provides tone, that is, in the space where the carer and the cared-for meet.^12^ According to Noddings,^13^ care ethics is based on the original description presented by Buber: An encounter may be considered as caring if one party acts as a ‘carer’ and the other one as the ‘cared-for’. Although not equal, both parties contribute to the establishment and maintenance of the caring relation. In care ethics, there is a strong emphasis on the difference between assumed needs and expressed needs.^13^ Tronto^14^ has further developed the ethics of care and found that there are four elements to consider: *caring about*, noticing the need to care; *caring for*, assuming responsibility for care; *caregiving*, the actual care that needs to be done; and *care-receiving*, the response from the cared for. The first element, *attentiveness*, refers to recognizing the needs of those around us and not ignoring them. The second, *responsibility*, is a central moral element of care ethics. The third, *competence*, refers to caregiving as a moral notion. The fourth element is the *responsiveness* of the care-receiver to the care given. All aspects of the ethics of care must be considered as part of an integrated whole based on the integrity of care.^14^ According to Mayeroff,^15^ to care for means relating to and responding to the entire person, not only some parts, by helping the other person reach self-realization and become responsible for her or his own life. Caring involves *trust* and *hope* and helping the other person to grow in her or his own time and way. An important aspect of hope is *courage*, that is, standing by the other person under trying circumstances and showing true responsiveness.

## Earlier research – the state of the art

A literature search was conducted using the following search terms: *school nurse*, *health promotion*, *health dialogue*, *care ethics*, *caring* and *adolescence*. The CINAHL and PubMed databases were searched, and manual searches were also conducted. A person-centred and professional approach in collaboration with a health team is an important aspect of health-promoting work that helps pupils engage in self-help, trust their own capabilities and become empowered. The emphasis is on encouraging, motivating and strengthening the involvement of adolescents in making their own decisions about their health.^16^ Being an attentive listener is an opener and enables the establishment of health-promoting dialogues. When the culture is permeated with knowledge, an authentic openness and a welcoming atmosphere with reflection are possible. The responsibility of confidentiality is highlighted as important for creating a sense of trust and security among adolescents.^8,17–19^ A welcoming atmosphere in combination with a caring school nurse who is professionally competent increases the possibilities of positive experiences and enhances health-promoting efforts. During health dialogues, school nurses should support adolescents in deriving the benefits of their own strengths and resources.^20–22^ Several studies shed light on the complexity of school nurses’ health-promoting work and the importance of creating a mutual and trustful environment. The school nurse should guide the adolescents and create a space for reflection which is the basis for change. Most health-promoting work involves collaboration with other professionals at schools and with parents, with the intention of developing and implementing joint strategies.^21,23^ The school nurse is supportive by providing advice and helping adolescents become aware of their own thoughts and feelings and create structure in their everyday life.^24–27^ The importance of the school nurses’ health-promoting work is evident in earlier research. The phenomenon of care ethics is implicit in the studies but not articulated. Therefore, this study sets out to highlight care ethics in the health-promoting activities of school nurses.

## Research aim

The aim of this study was to describe how care ethics is evident in the context of school nurses’ health-promoting activities among adolescents in secondary schools.

## Research design

An explorative descriptive methodology was chosen to identify recurring themes, patterns or concepts and, furthermore, to describe and interpret those patterns.^28^ An interview guide with open-ended questions was formulated. Individual interviews were used, in order to understand the phenomenon from the participants’ point of view.^28^

### Participants

All school nurses (N = 14) in a medium-sized municipality in Western Sweden, which is characterized by geographical and socioeconomic variety, were contacted by phone and invited to participate in the study. Two (n = 2) of the school nurses were excluded because they had recently started practising and lacked experience. Four (n = 4) school nurses declined the invitation. Finally, eight (n = 8) school nurses working at different secondary schools participated. All participants were women between the ages of 38 and 61 years. Their work experience as a registered nurse varied between 13 and 36 years, and all of them had been working as a school nurse for 5–18 years. Five participants had received specialist education as district nurses; two had double specialist education; and one had not received further education. The participants worked as school nurses between 60% and 100%, of their time and each of them was responsible for 150–550 pupils.

### Data collection

Individual interviews were conducted by G.P. and L.J. in October 2016. The school nurses chose a quiet place where the interview could be carried out undisturbed during working hours. The participants were encouraged to freely narrate their experiences and express their thoughts. An interview guide was used to guide the conversation and ensure objectivity. The conversation started with an open-ended question: Can you tell me about your health-promotion activities? The question was followed by subsequent questions to gain a deeper understanding: Can you tell me about health dialogue and your visions? What are the possibilities and potential challenges? The duration of the interviews varied between 45 and 60 min. All interviews were recorded and transcribed verbatim directly after the interviews.

### Data analysis

Data were analysed by content analysis.^29^ All transcribed interviews were read thoroughly by G.P. and L.J. to gain an understanding of the big picture. Following this, meaning units which expressed the thoughts and experiences of the participants were derived. In the first phase, the material was analysed independently by G.P. and L.J. In the second phase, they met several times to reflect upon the meaning units until they reached a common understanding. G.P. and L.J. also met with a senior researcher, Y.H., until they reached a consensus about the findings expressed in the main categories and sub-categories.

## Ethical considerations

This study was conducted in accordance with the ethical principles of the Swedish Research Council.^30^ According to Swedish law,^31^ there is no need for approval from an ethical committee to conduct a study among school nurses. Written and oral approval was obtained from the management officers at the schools where the data were collected. All participants were provided with both oral and written information about the study, as well as assurance about the confidentiality of their information and the option to withdraw from participation at any time. The participants were advised that the interviews were recorded and informed about the estimated duration of the interviews. All interviews took place during working hours, in the absence of any disruption. Participants signed a written consent form and were assigned individual codes (P1–P8) to ensure anonymity.

## Findings

The findings are presented as three main categories with sub-categories: engagement and caring for adolescents, collaboration and involvement of important stakeholders, and strong commitment to promoting adolescent health (Table 1).

**Table 1.** Examples of meaning units, sub-categories and main categories.

Meaning units	Sub-categories	Main categories
They need a huge amount of care, love…that you care and that you show that…I really care about you…	Genuinely caring about adolescents	Engagement and caring for adolescents
…the parents need to be involved in all health-promoting work…even if the pupil tells something difficult, I want the parents to take part…	Parental involvement	Collaboration and involvement of important stakeholders
The dream is to be able to have a health dialogue every year with all pupils regardless of grade…	Vision for improving health among adolescents	Strong commitment to promote adolescent health

## Engagement and caring for adolescents

The basic programme is based on a specific and regulated mission of school nurses, who are the only ones at school that meet all adolescents. Health dialogue is a tool via which the school nurses can identify adolescents’ specific needs and resources when planning health-promoting activities.

### Genuinely caring about adolescents

The participants talked about how they genuinely care about the adolescents at school and their well-being. One way of showing this was by being truly present in the encounter with the adolescents and building a trustful relationship. One characteristic of all participants was that they emphasized on and provided examples of how they really care about the adolescents.

They need a huge amount of care, love…that you care and that you show that…I really care about you. (P5)

They prioritized adolescents who do not have any adults to talk with or adults that they can trust. Having sufficient time for the conversation to develop and to end properly is important. Counselling and reflecting on the issues brought up by the adolescent is important, as the following quote demonstrates:

It’s very relevant and important for the pupil who comes wondering about something special…to meet and reflect on the thoughts of the pupil and ensure what the pupil should know when leaving. (P8)

In the interviews, being there for the adolescents was emphasized. They could visit the school nurse whenever and for whatever reason, ‘big things as well as small things’. Most school nurses do not have regular hours but are, instead, flexible and allow adolescents to visit when it suits them.

It is always open here – they may come anytime – they may come early in the morning or on their way home…They can always come…If I am here, I am here for the children…(P3)

It is important to stand up as an adult and to represent adults: ‘…being an adult and to stand up as an adult…this is something I miss among many parents today’ (P5). According to the participants, it is important for adults to make their presence felt in the school, by acting as role models whom adolescents can turn to.

There is a clear indicator in how the school nurses actively help adolescents to take care of themselves.

…it’s an important job to practice how to take care of oneself…to find ways…how does it feel good for you…it’s a kind of ‘the school of life’. (P1)

The purpose of health education is to help adolescents think for themselves and take responsibility for their actions:

It’s like a little advice to selfcare we try to convey to the pupils…that is what you are striving for. That they think for themselves and see their part. That they learn to take responsibility for their own well-being and knowing that there is help at hand and that they dare to ask for help. (P3)

### To observe and realize the unspoken

Participants narrated about the importance of the placement of their office. A favourable location is one with a view of the school yard, as this allows them to observe all the pupils during the day just by looking out the window. Thus, they could identify someone who looks sad, seems to be left out or is not feeling well, and quickly take action. It is also important that the school nurse show herself, by walking in the corridors that pupils occupy. The mere presence of adults in the school yard or in the corridors is considered to have a calming effect.

…that’s why I move in the corridors, I see and hear the pupils, what they are saying…It feels very natural…they come to me…‘I want to ask you something or I want to tell you something’…(P4)

Being an attentive listener who listens to what the adolescent says and also picks up on the unspoken was emphasized by the participants. Occasionally, an adolescent will visit the school nurse several times with some issues, when there actually might be another issue that is bothering them or something else that is not right with them.

…a pupil comes in…well, can I have a pain killer for my headache? Of course, I say…but tell me, have you had any breakfast today?…and depending on the answer you work from that…(P7)

It seems that adolescents try to cite another reason for visiting the school nurse if it is difficult for them to talk about the actual matter that bothers them. If the school nurse is listening and caring, these repeated visits for small things may lead to the adolescent opening up and gradually talking about the real problem.

### Importance of the health dialogue in health promotion

The basic programme is regulated and contains different types of screenings and vaccinations as recurring elements. Growth in terms of length and height is measured, back inspections are performed, and vision and colour sense are examined. Screenings are seen as duties that need to be ticked off the list and are described as actions taken to promote positive health.

In secondary school there is not much time for anything else than the basic program. However, there is a great need to discuss about nutrition, sleep and growth. (P2)

The health visits to the school nurse and the basic programme are offered to all adolescents, and usually, they all participate. Few do not participate, and it is usually because they forget about the designated time. The content of the health dialogue is aimed at growth, physical activity, food and sleep. However, the conversation is also about health issues in general, including well-being at home and at school and peer relationships. In secondary school, which is when adolescents start to explore more, the conversation is directed towards risk behaviour.

…we are talking about all sorts of things – with a focus on food and sleep and much about friends and relationships…about their well-being and if they are pleased with themselves. (P3)

The health dialogue is important because it is often during conversations that problems with sleep or physical activities emerge. When problem areas are identified, the conversation may be focused on further reflection around these matters.

We talk about sleep, and what happens in the brain when we are sleeping, why sleep is important…we talk about why it’s difficult to fall asleep if online on the mobile just before going to bed…and about being constantly available online…(P4)

Individual health dialogues offer one of the few possibilities of really listening to the thoughts of adolescents and having a conversation in peace and quiet. It gives adolescents the possibility of bringing up important questions and thoughts.

## Collaboration and involvement of important stakeholders

Collaboration between different stakeholders is important if health-promoting activities are to be successful. Good cooperation between teachers and other professionals at school, as well as the involvement of parents, is important.

### Health teams at schools

All participants expressed the importance of being a member of the school’s health team, through which several professions bear the responsibility for adolescent health. Some participants reported a lack of cooperation with teachers and, overall, a lack of interest in working together towards better health. However, most participants felt that the school nurse and the teachers complemented each other in the health-promoting work. Visiting classrooms and talking about health was an activity that the participants appreciated.

…the school encourages me to take initiatives and they are positive towards working in different ways with health-promoting activities. (P1)

The school nurses took advantage of all the possibilities of meeting with the adolescents and reflecting on health. Occasionally, the school nurse was contacted by teachers who needed his or her special knowledge and invited her to give a lesson on special topics that came up.

### Parental involvement

Another point that was emphasized was the involvement of parents and their interest in and responsibility towards the health of adolescents. All efforts towards gathering information and health education were considered to be in vain if the collaboration with parents did not work out.

…the parents need to be involved in all health-promoting work…even if the pupil tells something difficult, I want the parents to take part…(P1)

In the case of adolescents who are frequently absent from school, close collaboration with parents is required. Parents may be contacted, for example, in case of obesity or other physical problems. There may be a discussion with the parents about their thoughts and ways of collaboration to help their adolescent’s health.

…most common reason to have contact with the parents is when it concerns obesity and overweight…follow-up and maybe to motivate consultation with an obesity clinic…(P8)

Other reasons for a conversation might be contacting parents who have problems with speaking to their children. For example, parents might contact the school nurse when they face difficulties in handling the adolescent due to anger issues. In such cases, the school nurse can be supportive by discussing the setting of boundaries, among other things.

### Health activities to promote fellowship

The importance of participating in health activities arranged by the school is emphasized and seen as an important in showing engagement.

…I participate…as a school nurse if something happens, but also so that they see me out in the reality. I don’t want to be in a closed room, I want to participate…(P7)

Sports competitions may be arranged between different schools with the intention to build relationships, and to unite pupils and classes to strive towards a joint aim and to care for each other, that is, ‘to help the pupils to socially take care of each other’ (P8). School nurses meet with adolescents in groups, for example, with girls who show outspoken and disorderly conduct. They meet regularly and discuss confidentially how different issues can be sorted, and this has calming and positive effects. Some of the other mentioned health activities were lectures to counteract alcohol and drugs, and organization of so-called health weeks with themes about relationships.

## Strong commitment to promote adolescent health

Early intervention could help adolescents and, hence, hinder the deterioration of their health. Furthermore, there are many challenges with regard to adolescents with special needs, refugees, adolescents suffering from different diseases and those that do not attend school for various reasons.

### Vision for improving health among adolescents

The health dialogue is considered a perfect forum to meet with every single adolescent, to reflect on health and to identify possible problems and needs.

The dream is to be able to have a health dialogue every year with all pupils regardless of grade. (P7)

Annual health dialogues could provide school nurses with better conditions for health promotion via the development of a caring relationship with the adolescents, so that they can work together to identify risk factors, set up goals, and follow up and evaluate planned or implemented efforts. At present, they are often contacted too late, when the issue has already developed into a bigger problem. If they had been contacted earlier, they could have prevented the problem from occurring or becoming as severe. The participants also expressed a wish to work more in groups, as a group setting offers the opportunity to discuss and reflect on health and the factors promoting or hindering good health.

…we meet with all children separately every second year, and the second year we’d like to meet them in groups…we believe that it’s good for them to be able to discuss and reflect upon themselves and their health and things around it…(P6)

The participants believe that early interventions would make a great difference in terms of the availability of more resources for the health team as well as prioritization of collaboration with the parents, such as joint meetings, with the intention of strengthening their parental role.

### School absenteeism in adolescents

One of the greatest challenges is children who, for unknown reasons, do not attend school and sit at home instead. This group of students has grown large over the last few years, and there are records of adolescents who have not attended school for several weeks, months and, sometimes, years.

The greatest challenge is to reach out to all children who don’t feel well, those sitting at home, children who for some reason don’t go to school…this is a big challenge for the school. (P2)

Some participants were responsible for contacting the parents and arranging a home visit. In other schools, this responsibility lies with the headmaster and is seen as a pedagogic problem. The participants emphasized the necessity of early actions to tackle the issue of absenteeism from school.

…how shall we get them back to school?…it’s difficult if you start in the ninth grade, then it’s often too late…you have to start with early interventions. (P4)

In particular, it is difficult to tend to adolescents with a refugee background, because of the time it might take the school nurse to access all the documents concerning their health status. In addition, adolescents with neuropsychiatric diagnoses are also challenging as they need much support and help.

### Time limitations of health-promotion activities

There are many time-consuming routines that limit health-promoting activities. Early interventions are important for providing the right help at the right time, before the situation reaches a point where it is difficult to control. To facilitate early intervention, it is important to expand and/or reorganize the resources available to school nurses.

…hope that they would realize what it costs later on sometimes the focus is a little wrong…it is here and now and even earlier that it should be invested to promote good health. (P3)

The participants showed genuine engagement and commitment in caring for adolescents, and this is evident in their work on promoting health in a sustainable way.

## Discussion

In agreement with previous findings, care ethics in school nurses’ health-promoting activities becomes evident in their strong commitment towards and engagement in caring for and caring about the adolescents.^12–15^ In addition, there was emphasis on creating a trustful and supportive caring relationship by promoting adolescents’ engagement and their sense of belonging, and encouraging meaningful involvement.^32^

The school nurses underline the importance of being an attentive and good listener, by listening to both the spoken and unspoken, that is, the expressed feelings and experiences of adolescents. If school nurses are open and observing, the starting point will be ‘expressed needs’ instead of ‘assumed needs’. In the encounter, the school nurse responds to the expressed needs of the adolescent who, in turn, responds and becomes actively involved.^13–15^ Such a caring and trustful relation creates the space for a supportive health dialogue with reflection, which is the basis for change and empowerment. Accordingly, flexibility, keeping a low threshold and caring for the adolescents are also mentioned in several earlier studies.^17–21,32^ However, school nurses experience ethical conflicts because they acknowledge the expressed needs of adolescents but do not have enough time for individual health dialogues or group conversations.^33,34^ The basic programme with screenings is time-consuming and poses a limitation to health-promotion activities. In fact, a task-centred approach is inconsistent with the principles of person-centred care.^1,6,7^ In agreement with the present findings, an excessive workload with stipulated screenings has been found to prevent school nurses from realizing their ideals and performing health-promoting activities.^21,34,35^ Many early interventions may be missed out and symptoms may develop into severe problems that affect health and well-being before the school nurse is contacted. The school nurses believe that they would be able to help adolescents via early interventions if they are contacted in time. School nurses experienced moral stress when they were unable to use their competence and initiate early interventions in time.^21,33,35^

Good teamwork in collaboration with parents, teachers and other professionals in the health team is considered very important. The involvement of the entire health team in handling complex and demanding situations and ethical reflections might support school nurses in their endeavour.^36^ Collaboration with parents is highlighted, since health habits are carried into adulthood.^21,23,37^ However, a non-supportive work environment where some teachers are not interested in health issues or disorganization of work can cause ethical conflicts.^21,34,35^

Adolescents have, in general, a healthy lifestyle, but the incidence of sleep problems, stomach pain, headaches, stress and depression has increased.^1–3^ These are not medical diagnoses but, rather, symptoms that adolescents may need support and guidance to manage in their everyday life. The present findings show that school nurses are guided by their values and competence in empowering adolescents through self-help and taking on the responsibility of their own lives and health. They genuinely *cared about* and *cared for* the adolescents due to their values and ideals, which were evident through their action. The character of the school nurse is determined by the ethical foundation, the motives for caring and responsibility, and their will and interest in inviting and welcoming the adolescent to a caring relation. School nurses who are in touch with their innermost core, or ethos, have the courage to take on responsibility and to be engaged. In addition, the values of the school nurse are reflected in the physical room where the encounter takes place.^11–15^

In a recent editorial, the question ‘*Should 2020 be the decade of character in care*?’ was asked.^38^ This study provides evidence for the significant meaning of care ethics and the character of school nurses as the basis for successful health promotion among adolescents. The necessary ethical guidelines were followed and the transparency of information was ensured in the conduct of this study.

### Limitations of the study

A major limitation is the sample size, as only eight school nurses participated. Furthermore, the participants were all female because no male school nurses worked in the municipality. However, small sample size does not affect the credibility and trustworthiness of a study, if the interviews are prepared, conducted and analysed well.^29^ The school nurses had extensive and solid experience and provided rich material, which guarantees the trustworthiness of the study.

## Conclusion

Strengthening person-centred healthcare can provide adolescents with the recognition and support they need to grow into healthy adults. For successful health promotion, all aspects of the ethics of care should be considered as part of an integrated whole based on the integrity of care. An education with a solid value base of care ethics, as a moral notion, is needed if the intention is to have committed school nurses with an inner ethical stance and a strong caring identity. Further research is needed on care ethics and caring relations where adolescents are engaged in the ownership of health and positive change.
